# Explainability of Protein Deep Learning Models

**DOI:** 10.3390/ijms26115255

**Published:** 2025-05-29

**Authors:** Zahra Fazel, Camila P. E. de Souza, G. Brian Golding, Lucian Ilie

**Affiliations:** 1 Department of Computer Science, University of Western Ontario, London, ON N6A 5B7, Canada; 2 Department of Statistical and Actuarial Sciences, University of Western Ontario, London, ON N6A 5B7, Canada; 3 Department of Biology, McMaster University, Hamilton, ON L6S 4K1, Canada; golding@mcmaster.ca

**Keywords:** protein interactions, protein embeddings, XAI methods

## Abstract

Protein embeddings are the new main source of information about proteins, producing state-of-the-art solutions to many problems, including protein interaction prediction, a fundamental issue in proteomics. Understanding the embeddings and what causes the interactions is very important, as these models lack transparency due to their black-box nature. In the first study of its kind, we investigate the inner workings of these models using XAI (explainable AI) approaches. We perform extensive testing (3.3 TB of total data) involving nine of the best-known XAI methods on two problems: (i) the prediction of protein interaction sites using the current top method, Seq-InSite, and (ii) the production of protein embedding vectors using three methods, ProtBERT, ProtT5, and Ankh. The results are evaluated in terms of their ability to correlate with six basic amino acid properties—aromaticity, acidity/basicity, hydrophobicity, molecular mass, van der Waals volume, and dipole moment—as well as the propensity for interaction with other proteins, the impact of distant residues, and the infidelity scores of the XAI methods. The results are unexpected. Some XAI methods are much better than others at discovering essential information. Simple methods can be as good as advanced ones. Different protein embedding vectors can capture distinct properties, indicating significant room for improvement in embedding quality.

## 1. Introduction and Background

The field of computational biology has undergone significant transformations in recent years, driven mainly by the advent of deep learning models. Deep learning approaches have provided unprecedented insights into protein structure prediction, function annotation, and interaction dynamics, enabling researchers to explore the complexities of biological systems with remarkable precision and efficiency [1,2,3,4].

Deep learning models have emerged as powerful tools in this context, capable of processing vast amounts of biological data and extracting meaningful patterns that often elude human perception. These models have demonstrated remarkable success in predicting protein structures, identifying functional sites, and elucidating protein–protein interactions. Such capabilities have far-reaching implications for various fields, including drug discovery, personalized medicine, and synthetic biology [1,5,6,7].

Despite these advancements, the application of deep learning in computational biology has its challenges. One of the most pressing concerns is the potential for errors in the predictions of these models. A significant factor contributing to this challenge is the black-box nature of deep learning models. These models often transform inputs into outputs through complex, multi-layered neural networks, with little insight into the intervening processes. The lack of interpretability raises concerns about the reliability and trustworthiness of these models in high-stakes biological applications.

In this paper, we address the need for enhanced explainability in deep learning models applied to protein analysis. We explore methods to elucidate the decision-making processes of these models. This paper aims to bridge the gap between complex computational techniques and their practical applications in biology. Our goal is to foster a deeper understanding of how these models work, ultimately leading to more accurate predictions, better scientific discoveries, and safer, more practical applications.

We address two specific problems, protein embedding construction and protein interaction-site prediction, which we attempt to clarify using methods for AI explainability. We introduce these topics below.

### 1.1. Protein Embeddings

The natural language processing (NLP) field has undergone a revolutionary transformation through the emergence and development of contextual embeddings. This journey began with early models like Word2Vec [8] and GloVe [9] and moved to more sophisticated context-dependent architectures such as BERT [10] and T5 [11]. A critical factor in this progress has been the technique of self-supervised learning. This approach allows models to extract meaningful representations from vast amounts of unlabeled data, eliminating the need for extensive manual annotation.

Inspired by the remarkable success of these approaches in NLP, researchers have applied similar principles to the domain of proteomics. In this field, protein residues (amino acids) are associated with high-dimensional numerical vectors analogous to word embeddings in NLP. This cross-disciplinary transfer of knowledge has led to the development of a diverse array of protein embedding models. These include but are not limited to ProtVec [12], SeqVec [13], SSA [14], MSA-transformer [15], ProtBert, ProtT5 [4], ESM2 [16], and Ankh [17]. Each model brings unique strengths and capabilities to the table, contributing to the growing toolkit available to researchers in proteomics.

The impact of protein embeddings is far-reaching, as they enable researchers to gain deeper insights into the complex relationships between protein sequences, structures, and functions. This enhanced understanding can pave the way for advancements in protein engineering, drug discovery, and the elucidation of cellular mechanisms, ultimately contributing to the overall progress in the life sciences. In this paper, we investigate explanations for the construction of such embeddings for three methods: ProtBERT, ProtT5, and Ankh. Other interesting candidates include ESM2, another top method; SSA, a biLSTM-based method; and MSA-transformer, an alignment-based method. However, GPU memory limitations imposed constraints on the length of the proteins we could test (see the Results section). ESM2 and MSA-transformer take a batch of protein sequences as input, which means that the length of the tested proteins should be even smaller.

### 1.2. Protein Interaction-Site Prediction

The other problem we consider concerns a fundamental issue in proteomics: protein interaction-site prediction. Proteins are among the most important molecules in a cell and are responsible for a multitude of essential processes, which they perform by interacting with other proteins. Therefore, the investigation of these interactions is a key problem. Protein interaction-site prediction aims to identify the specific regions of a protein where interactions with other molecules are most probable. Recent developments in protein embeddings, as mentioned above, have helped to significantly improve the state of the art in interaction-site prediction. Various deep learning models have been developed to tackle this challenge, including GraphPPIS [18], DeepPPIS [19], DLPred [20], DELPHI [21], PITHIA [22], ISPRED-SEQ [23], and Seq-InSite [24]. These models can be divided into two main categories: structure-based and sequence-based. Structure-based models use 3D structures of proteins as input, while sequence-based models use protein sequences as input. Seq-InSite [24] is the current state-of-the-art model for this task, having outperformed structure-based models despite being sequence-based. We look into explaining how Seq-InSite makes its predictions.

### 1.3. Explainable AI (XAI) Methods

Explainability is the degree of human comprehension of an AI model’s decision-making processes and inherent biases. It encompasses methodologies that elucidate the internal mechanisms of AI systems, fostering user understanding and trust. The pursuit of explainability serves multiple critical functions: it enhances transparency, facilitates debugging, enables model improvements, and promotes knowledge discovery from otherwise opaque “black-box” models [25]. Three categories of explainability methods are investigated in this work: gradient-based, path-attribution, and local model-agnostic methods.

Gradient-based methods are designed to determine the gradient of a prediction or classification score relative to the input features. The differences between the methods in this category depend on how these gradients are calculated [25]. Saliency maps (Vanilla Gradient) [26] compute the gradient of the class score of interest with respect to the input pixels by approximating the score with a first-order Taylor expansion. A deconvolutional network (DeconvNet) [27] can be seen as a convolutional neural network (ConvNet) operating in reverse. This method inverts the forward pass of a traditional ConvNet, enabling the visualization and interpretation of learned features at various network layers. Guided backpropagation [28] integrates both saliency map and DeconvNet approaches and selectively filters gradients during backpropagation using a modified version of the backpropagation algorithm. Input X Gradient [29] calculates attributions by multiplying the derivatives of the output with respect to the input element-wise with the input itself. This approach combines the sensitivity information captured by gradients with the scale of the input features, potentially offering more precise and interpretable attributions than previous gradient-based methods [30].

Path-attribution methods analyze the influence of an input on a model’s prediction by contrasting it with a reference point. By evaluating the difference between the predictions for the original input and the reference input, these methods distribute this difference across all features of the original input [25]. DeepLIFT [31] assigns importance scores to input features based on differences in neuron activations compared to a reference. It overcomes gradient discontinuities and provides non-zero contributions even when gradients are zero. Integrated Gradients [32] is based on two fundamental principles of attribution methodologies: sensitivity and implementation invariance. The former stipulates that features receive non-zero attribution if and only if they are influencing predictions, while the latter requires that functionally equivalent networks yield identical attributions.

Local model-agnostic methods explain individual predictions [25]. LIME (Local Interpretable Model-agnostic Explanations) [33] approximates the model locally around an instance of data by first generating perturbed samples of that instance and then weighing them based on proximity. Then, it trains an interpretable model like a decision tree on these samples and uses it to explain the instance’s prediction. SHAP (Shapley Additive Explanations) [34] is based on the Shapley values from game theory. This method considers features’ importance as players in a coalition, with the model’s predictions as the payout. SHAP uses Shapley values to allocate this payout among features fairly. KernelShap [34] is an extension of SHAP that integrates LIME with a linear explanation model and Shapley values. GradientShap [35] offers an alternative approach for estimating SHAP values based on two key assumptions: the independence of input features and the linearity of the explanation model.

**Figure 1:**
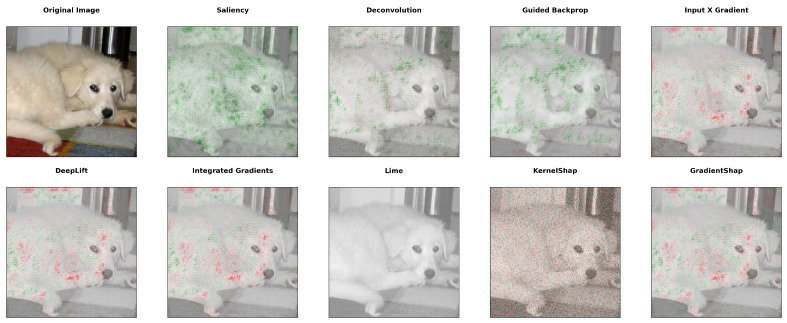
Output of explainability methods for a CNN classification model on the same image. Green pixels have a positive contribution, while red ones have a negative impact on predicting the label of the picture.

To summarize, the methods we employ are saliency maps, deconvolution, guided backpropagation, Input X Gradient, DeepLIFT, Integrated Gradients, LIME, KernelShap, and GradientShap. Different methods can provide very different explainability results, as seen in the example in Figure 1.

### 1.4. Explaining Protein Learning Models

**Figure 2:**
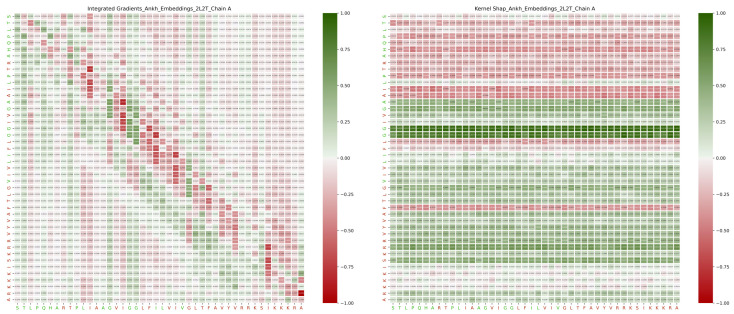
Explainability example: ProtT5 embedding computation for protein sequence 2L2T_A, explained using Integrated Gradients (**left**) and KernelShap (**right**). On the left and in the bottom margins, interacting residues are shown in green, and non-interacting ones in red.

Despite the wide range of applications of transformers, limited research has been carried out on their explainability beyond visualizing their attention layers [36,37,38,39]. To the best of our knowledge, this is the first investigation into the explainability of protein language models. We consider the embedding construction and interaction-site prediction for three embedding methods using nine XAI methods for 34 protein sequences that fulfilled the requirements. For a given protein sequence of length *n* and any given XAI method, the explainability matrix (see Methods) is an n×n matrix, whose (i,j) entry gives the effect, or influence, of the $j^{\mathrm{th}}$ residue in computing the embedding vector (or prediction value) of the $i^{\mathrm{th}}$ residue. Figure 2 gives two such matrices for the same protein, 2L2T_A, and embedding method, ProtT5, which are very different due to the different XAI methods used.

The evaluation of explainability is performed in several ways. After ensuring that our matrices are fully distinguishable from random, we first calculate the correlation between the explainability matrices and each of the seven amino acid properties: interactivity, aromaticity, acidity/basicity, hydrophobicity, molecular mass, van der Waals volume, and dipole moment. Next, we evaluate how each XAI method works on influencing residues according to the distance separating them. Finally, the infidelity measure is used to evaluate the quality of the explanations of each method.

## 2. Results and Discussion

### 2.1. Data

We used a modified dataset of protein sequences from the one used by Seq-InSite [24]. Due to the limitations of the Captum library [35], it is not possible to perform explanation methods on one sequence using multiple GPUs. Therefore, GPU memory limitations meant that our experiments had to use protein sequences with at most 44 residues. We selected the protein sequences, as shown in Table 1, removed them from the training data, and retrained Seq-InSite with the remaining protein sequences.

**Figure 3:**
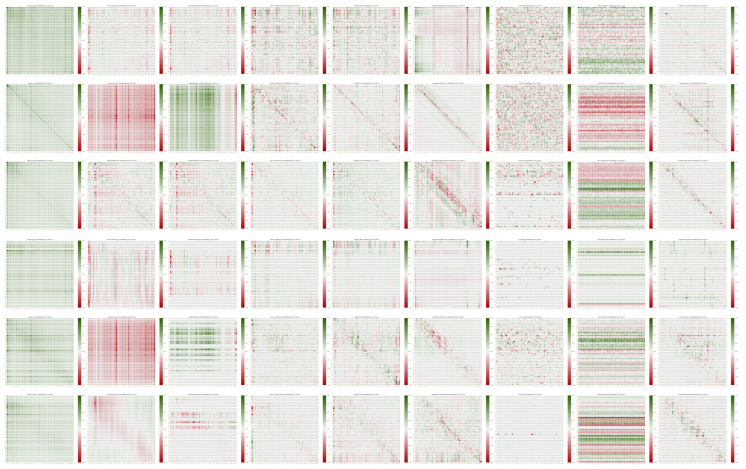
Interpretations for protein 2L2T_A. The first three rows are for embeddings—ProtBERT, ProtT5, Ankh—and the next three are for Seq-InSite predictions using the three embeddings, in the same order. From left to right we have the nine XAI methods in the order mentioned above.

For each protein, we computed explanation matrices for three embedding methods—ProtBERT, ProtT5, and Ankh—and for Seq-InSite predictions using the three embeddings as part of its input. We applied the nine XAI methods mentioned above. This resulted in a total of 54 explanation matrices for each protein sequence. All the matrices for the 2L2T_A protein are shown in Figure 3. A wide variety of patterns can be seen across the embeddings and XAI methods. Diagonal, row-wise, and column-wise patterns are visible, together with more complex patterns, even for the same method or the same test type.

### 2.2. Comparison with Random Matrices

In order to demonstrate that the resulting explanations were not arbitrary and contained relevant information, we utilized an SVM classifier with a Radial Basis Function (RBF) kernel for training on 80% of the given explanations. The trained classifier was then evaluated on the remaining 20% of the explanations. The classifier accurately distinguished between explanation maps and random matrices with a 100% accuracy rate. This indicates that a hyperplane separates these explanations from random matrices with a margin, ensuring a clear separation and validating the information content within the explanations.

### 2.3. Amino Acid Properties

We investigated seven properties of amino acids, which are briefly described below. Three such properties are categorical—interactivity, aromaticity, and acidity/basicity—while the other four are numerical—hydrophobicity, molecular mass, van der Waals volume, and dipole moment. They are summarized in Table 2 with the exception of interactivity, which is dataset-dependent. For all protein sequences in Table 1, the interaction sites are known. The goal of the Seq-InSite program is to predict these interaction sites as accurately as possible.

*Interactivity* is the property of each residue to interact with other proteins. *Hydrophobicity* denotes the propensity for certain residues to minimize interactions with aqueous environments, preferentially associating with non-polar substances. This property significantly influences protein folding, stability, and intermolecular interactions [40]. The *molecular mass* of amino acids is defined as the cumulative sum of the atomic masses comprising a single amino acid molecule and serves as a fundamental parameter in various biochemical and analytical procedures [41]. The *van der Waals volume* quantifies the spatial occupation of an atom or molecule, encompassing the region influenced by its electron cloud, and is crucial for elucidating molecular interactions and steric effects [41]. The *dipole moment* represents a vectorial quantity characterizing the magnitude and orientation of charge separation within the molecular structure and plays a significant role in determining the electrostatic properties and intermolecular interactions of amino acids [41]. The *aromaticity* of amino acids is determined by the presence of a six-carbon ring in the side chain; phenylalanine (F), tyrosine (Y), and tryptophan (W) are classified as aromatic [40]. The *acidity*/*basicity* is determined by whether the amino acids exhibit acidic properties (aspartic acid (D) and glutamic acid (E)) or basic characteristics (histidine (H), lysine (K), and arginine (R)) [40].

For each of these properties, we calculated the mean explanation score for each of the 20 amino acids (see the Methods section). Explainability provides the impact that each input residue—called the *source*—has on the value (embedding or prediction) of any output residue—called the *target*. The calculation of the mean explanation score for each of the 20 amino acids was performed separately for the source and target. Therefore, for a given embedding and XAI method, we had four tests for each property, combining embeddings/predictions with source/target.

### 2.4. Comparison of Amino Acid Properties

In order to evaluate how well an explanation captures an amino acid property, we used the Mann–Whitney U test for each of the three categorical properties and Kendall’s τ test for the four numerical properties. The results for the categorical tests are given in Table 3 for ProtBERT, Table 4 for ProtT5, and Table 5 for Ankh. The results for the numerical tests are given in Table 6, Table 7 and Table 8. In all cases, *p*-values below 0.05 are considered significant and shown in boldface. Kendall’s correlations in Table 6, Table 7 and Table 8 are also provided as a heat map for better visualization.

The statistical tests for the categorical properties appeared to be easier to pass than those for the numerical properties: 53% of the former tests were passed overall, whereas only 18% of the latter tests were passed. Overall, one-third of the tests were passed. Besides the sparsity of significant *p*-values in Table 6, Table 7 and Table 8, one can see a number of disagreements between the XAI methods, with the same property providing a positive correlation for one method but a negative correlation for another; a good example is the dipole moment in Table 6, where Integrated Gradients exhibits a positive correlation, with significant *p*-values in three out of four cases, whereas saliency exhibits a negative correlation, with all four cases having significant *p*-values. The largest difference is found in Table 7, also for the dipole moment, in the top quarter between saliency and KernelShap. Such cases are present in both the embedding and prediction tables for all three embedding methods, albeit mostly with non-significant *p*-values. The reason for this may be related to the possible instability in how embeddings represent physicochemical properties or the variability among XAI methods.

A summary is presented in Table 9. In terms of the XAI methods, KernelShap performed the best, with 45 tests passed, followed, unexpectedly, by the simple saliency method with 41, and guided backpropagation with 37. The last column of Table 9 indicates a wide range of performance, with LIME performing the worst, with nine tests passed.

Regarding embeddings—bottom row of Table 9—things were well balanced, with the three methods performing similarly overall, with 78, 84, and 86 tests passed. However, there was a significant difference between the categorical tests, where Ankh performed the best, and the numerical tests, where ProtT5 performed the best. ProtBERT performed second best in both and was slightly behind overall.

Breaking down the results by XAI method and test type, as shown in Table 10, there appears to be a good balance most of the time between the target and the source, as well as between the embedding and the prediction, with the exception of GradientShap and, especially, LIME. Interestingly, LIME, which ranked last overall, performed the best in the target numerical tests and prediction numerical tests. In fact, eight out of nine tests passed by LIME were in the prediction and target numerical tests.

Breaking down the results by embedding and test type, as shown in Table 11, there appears to be a better balance among the performance of all embeddings across the four test types, with source and embedding generally lagging behind target and prediction. Overall, with the notable exception of LIME, the passed tests were fairly well distributed across the different categories.

**Figure 4:**
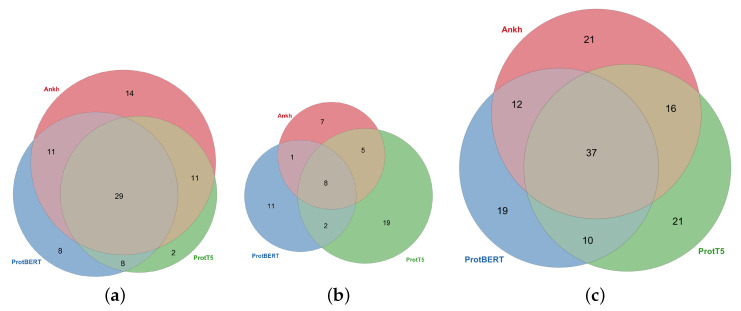
Embedding comparison for (**a**) categorical tests, (**b**) numerical tests, and (**c**) all tests.

It is important to note that, even though the number of tests passed by the three embeddings was similar, the embeddings actually performed very differently, and complementarily, for different tests. In Figure 4, we plot the situation for categorical, numerical, and all tests separately. In the categorical tests, 14 tests were passed only by Ankh, while in the numerical tests, 19 tests were passed only by ProtT5. A large proportion of the tests were passed by only a single embedding method: 22% for categorical and 70% for numerical, with 45% overall. This result is important as it indicates a large potential for improvement in the embedding generation.

Another observation concerns the fact that Seq-InSite uses a given embedding to produce its predictions. Therefore, it can predict certain properties only when the necessary information is available in the embedding itself. This means that, for a test, given the embedding and XAI method, it is unexpected for the prediction to pass the test while the embedding fails. Table 12 summarizes all four possible situations, combining whether the embedding was a pass/fail with the prediction being a pass/fail. There were fewer unexpected fail/pass situations for the categorical tests than for the numerical ones. ProtBERT had the most, and ProtT5 had the fewest.

The reason for the existence of these fail/pass cases is unclear. The property must be available in the embedding to be picked up by Seq-InSite; therefore, it is possible that the XAI methods sometimes failed to identify it at the embedding stage. It is interesting to note that the number of fail/pass cases appears to be inversely proportional to the performance of the embeddings for protein interaction-site prediction; ProtT5 performed the best, with Ankh ranking second and ProtBERT last. On the other hand, the performance ranking is not completely reliable, as Ankh passed slightly more tests overall than ProtT5, as shown in Table 9. More testing is needed to clarify what caused this behavior.

### 2.5. Distances

**Figure 5:**
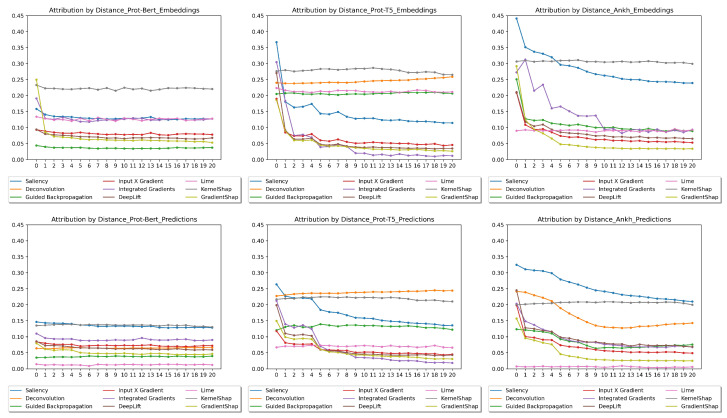
Distance plots: The average attribution between residues in terms of the distance between them. The embedding tests are shown in the **top row**, and the prediction tests are shown in the **bottom row**. From left to right are the results for ProtBERT, ProtT5, and Ankh.

It is expected that a residue in close proximity to another exerts a more significant influence on its interactions than those located further away. For each explanation method and both embeddings and predictions, we conducted an analysis to determine the average impact score for amino acids separated by a fixed distance. The resulting impact scores are plotted as a function of the distance in Figure 5. While the expectation is that the influence diminishes with distance, the hope is that the influence maintains a high level even at longer distances. Note that, in the ProtBERT and Ankh embedding distance plots, deconvolution and guided backpropagation have the same values; therefore, the line for deconvolution is covered by the one for guided backpropagation.

The first observation regarding the plots in Figure 5 is that the influences were generally smaller for all XAI methods in the case of ProtBERT (first column). Overall, KernelShap, saliency, guided backpropagation, and deconvolution were among the best-performing methods, which is in agreement with our results for test passing. One exception to this was the poor performance of guided backpropagation and deconvolution for ProtBERT. KernelShap and saliency were still the top two methods for ProtBERT. LIME, the last-ranked method overall in test passing, exhibited mixed performance in terms of the distance plots, ranking last in two cases out of six. GradientShap, the second-last-ranked method overall in test passing, exhibited very poor performance in terms of the distance plots. In general, there was good agreement between the distance plots and the previous results for test passing.

### 2.6. Infidelity

Infidelity measures the quality of explanation as the ability of an XAI method to capture changes in a prediction in response to perturbations (see the Section 3). We present the infidelity results in Table 13. While there are some differences among the XAI methods for a fixed embedding method (ProtBERT, ProtT5, or Ankh) and mode (embedding vs. prediction), the largest differences appear when the embedding or mode changes. That is, the infidelity values appear to be dictated mostly by the embedding method and the mode. Using Ankh appears to yield the lowest infidelity values overall. To investigate the correlation between the infidelity values and our results for test passing, we present the latter in Table 14, organized in the same way as in Table 13. While there is some correlation, important differences stand out. Several saliency infidelity results are high (which is bad) despite the number of tests passed being very high. Conversely, LIME achieved mostly very good infidelity values, while its test-passing performance was very low. Overall, the correlation between the infidelity results and test passing was not very good. The possible reasons for this are numerous and remain to be investigated.

## 3. Materials and Methods

### 3.1. Interpretability of Protein Embeddings

For each residue, an embedding vector of size *e* is constructed using a method such as the ones investigated here: ProtBERT, ProtT5, and Ankh. ProtBERT and ProtT5 produce embedding vectors of size e=1024, while Ankh’s vectors are of size e=768. The input for a protein language model is a protein sequence, and its length is denoted by *n*. The output of the model is an embedding matrix of size n×e, one vector for each residue. The first layer in the model is an embedding layer, a look-up table that outputs an n×e matrix. Since this layer is not trainable, it does not have a gradient; therefore, it is not interpretable. The interpretation connects this layer with the output, computing the influence (attribution) for each element on each element of the output, thus producing an n×e×n×e array, denoted by *E*; the element E[i,k,j,ℓ] gives the effect of the input (j,ℓ) on the output (i,k). To obtain the effect of one residue on another residue, with respect to embedding computation, we convert this array to an n×n array by calculating the sum along the second and fourth dimensions:

$$X_{E}\left[i,j\right]=\sum_{k=1}^{e}\sum_{\ell =1}^{e}E\left[i,k,j,\ell \right]\;.$$

The array $X_{E}$ measures the impact of each residue on computing the embedding vector of any other residue. The element $X_{E}\left[i,j\right]$ gives the effect of the $j^{\mathrm{th}}$ residue on computing the embedding vector of the $i^{\mathrm{th}}$ residue. Examples are shown in Figure 2 and in the top three rows of the plots in Figure 3.

### 3.2. Interpretability of Interaction-Site Prediction Models

Seq-InSite uses the embedding vectors of the residues within a window of an odd size, 2w+1, centered on the residue being predicted. Therefore, it takes as input a (2w+1)×e matrix and returns a number between 0 and 1. To interpret the interaction-site prediction for a sequence with *n* residues, we iterate over the sequence and obtain the interpretation of the model’s output for each residue. Therefore, the interpretation output is an n×(2w+1)×e matrix, denoted by *P*. The element P[i,j,k] gives the effect of the $k^{\mathrm{th}}$ element of the embedding vector of the $j^{\mathrm{th}}$ residue on the prediction of the $i^{\mathrm{th}}$ residue. In order to obtain the effect of each residue on the prediction of any other residue, we need to combine this array *P* with the *E* array above. Recall that *E* gives the effect of input embedding vector elements on output embedding vector elements. To obtain the effect of residues on embedding vector elements, we sum along the fourth dimension:

$$T\left[i,j,k\right]=\sum_{\ell =1}^{e}E\left[i,j,k,\ell \right]\;.$$

*T* has dimension n×e×n and T[i,j,k] gives the effect of the $k^{\mathrm{th}}$ residue on computing the element (i,j), that is, the $j^{\mathrm{th}}$ element of the embedding vector for the $i^{\mathrm{th}}$ residue. In order to put together *P* and *T*, note that, for each residue *i*, we need only a slice of size 2w+1 of *T*, T[i−w..i+w,:,:]. The n×n array $X_{P}$, for residue on residue impact for interaction prediction, is computed as follows: for any 1≤i,j≤n, we have:

$$X_{P}\left[i,j\right]=\sum_{k=1}^{2w+1}\sum_{\ell =1}^{e}P\left[i,k,\ell \right]\times T\left[i+k-\left(w+1\right),\ell ,j\right]\;.$$

The array $X_{P}$ gives the residue-on-residue impact for prediction. Precisely, $X_{P}\left[i,j\right]$ gives the effect of the $j^{\mathrm{th}}$ residue on the interaction propensity for the $i^{\mathrm{th}}$ residue. Examples are shown in the bottom three rows of the plots in Figure 3.

### 3.3. Evaluation of Interpretations

#### 3.3.1. Categorical Tests

For the categorical tests, we used the Mann–Whitney U test [42], which is a non-parametric statistical test for analyzing the distribution of two random variables, with the null hypothesis that the distributions are equal. In order to evaluate the performance of the models in capturing specific properties, this test was applied to both embeddings and predictions, focusing on the impacting (source) and impacted (target) scores of three distinct groups:

1. Interacting and non-interacting amino acids.
2. Aromatic and non-aromatic amino acids.
3. Acidic and basic amino acids.

If the models effectively captured these properties, the Mann–Whitney U test should reveal a significant difference between the distributions of the two populations within each group. *p*-values below 0.05 are considered significant.

#### 3.3.2. Numerical Tests

For the numerical tests, we used Kendall’s τ test, another non-parametric statistical test based on the correlation between two random variables, with the null hypothesis that there is no correlation between the random variables. To assess the effectiveness of each explanation method, we computed the average source and target scores of each amino acid across all proteins for both embeddings and predictions. This process yielded four distinct vectors, each of size 20. Subsequently, for each of these four vectors, we employed Kendall’s τ test to investigate the correlation between the vector and various properties of amino acids: hydrophobicity, molecular mass, van der Waals volume, and dipole moment. The underlying assumption is that a correlation should be evident if the model accurately captures the specific property under consideration. *p*-values below 0.05 are considered significant.

#### 3.3.3. Distance

From a biological perspective, the average interaction of each residue, ignoring its three-dimensional location, is more influenced by its linear distance from nearby residues than by those farther away. Therefore, for each explanation method, and for both embeddings and predictions, we computed the average impact score for amino acids separated by a distance of i,0≤i≤20, and plotted these scores against the distance. The expectation is that these plots exhibit a decreasing trend.

#### 3.3.4. Explanation Infidelity

One way to evaluate the quality of an explanation is to measure how much it captures the changes in the predictor function in response to significant perturbations. Explanation infidelity is defined as follows [43]. Let $X\subseteq R^{d}$, Y⊆R, and let $f:R^{d}\to R$ be the input space, output space, and a black-box predictor, which at some test input $x\in R^{d}$ predicts the output f(x). Let $\Phi :F\times R^{d}\to R^{d}$ be a feature attribution explanation that, given a black-box predictor, *f*, and a test input, *x*, returns importance scores Φ(f,x) for the set of input features. Given a random variable $I\in R^{d}$ with a probability measure $\mu_{I}$, which represents the meaningful perturbations of interest, the explanation infidelity of Φ is defined as

$$\mathrm{INFD}\left(\Phi ,f,x\right)=E_{I∼\mu_{I}}\left[{\left(I^{T}\Phi \left(f,x\right)-\left(f\left(x\right)-f\left(x-I\right)\right)\right)}^{2}\right]\;.$$

In our tests, a normal distribution with a mean of 0 and a standard deviation of 0.01 was used as a perturbation and applied to the outputs of the embedding layer of the transformer model and to the embedding inputs of the prediction models.

The formula for INFD above was used on each matrix E[i,j,:,:], for 1≤i≤n,1≤j≤e, to produce an infidelity score. This resulted in an n×e matrix, $I^{E}$, where $I_{ij}^{E}$ is the infidelity score for the interpretability of element (i,j) in the embeddings. The infidelity score for the whole interpretation of embeddings is defined as the mean of $I^{E}$:

$${\mathrm{INFD}}^{E}=\frac{\sum_{i=1}^{n}\sum_{j=1}^{e}I_{ij}^{E}}{ne}\;.$$

To calculate the infidelity scores for the Seq-InSite model interpretations, the above formula for INFD was used on each matrix T[i,:,:] to give a vector $I^{S}$ of size *n*. Then, the infidelity of the prediction interpretations was calculated as follows:

$$\begin{array}{ccc} {\mathrm{INFD}}^{P} & = & \frac{S}{\left(n-2*w\right)\left(2w+1\right)+\left(\sum_{i=w+1}^{2w}i\right)}\\ & = & \frac{S}{\left(n-2w)(2w+1)+w(3w+1\right)} \end{array}$$

where

$$\begin{array}{ccc} S & = & \left(\sum_{i=w+1}^{n-w}\sum_{j=1}^{e}I_{ij}^{E}+\sum_{i=w+1}^{n-w}I_{i}^{P}\right)\times \left(2w+1\right)\\ & & +\sum_{i=1}^{w}\left(\left(\sum_{j=1}^{e}I_{ij}^{E}+\sum_{j=1}^{e}I_{(n-i+1)j}^{e}+I_{i}^{p}+I_{n-i+1}^{p}\right)\times \left(w+i\right)\right)\;. \end{array}$$

### 3.4. Implementation

The tests were performed using Python 3.10.0, Hugging Face transformers 4.31.0 [44], PyTorch 2.0.1 [45], and the Captum library 0.6.0 [35] to calculate and analyze protein embeddings. Because the attribution can be calculated for only one element at a time, computing the explanation for one protein sequence took between one and five days, depending on the protein sequence, embedding model, and method. They were computed once and stored. Due to the n×e×n×e size of the initial arrays, the total memory required to store all our computed matrices was 3.3 TB.

## 4. Conclusions

In this work, we performed extensive experiments to understand protein deep learning models through their explanations. Based on these experiments, we showed that protein language models comprehend seven important biological characteristics of amino acids. We also demonstrated that no single explanation method consistently performed best across all models and metrics and that they showed high variability in performance across different models. While current explanation methods work for short proteins, improvements are needed to make them applicable to long proteins, which are more common in real-world scenarios.

We showed that the direct evaluation of explanations provided by the infidelity measure was not consistent with the indirect evaluation, as indicated by the ability to capture various physicochemical properties. The reason for this is unclear and remains to be investigated.

Our investigations showed a very complex picture. We raised more questions than we answered. More work is needed to clarify the findings. Longer protein sequences have to be tested, as explained above, for more reliable results. Also, more embedding methods have to be tested, as well as more prediction problems. All models discussed—regarding embedding generation, prediction, and explanation—are very complex, and enhancing our understanding of them is both important and difficult.

## Figures and Tables

**Table 1:** Protein sequences used for testing.

Protein ID	Chain	Length	Protein ID	Chain	Length
2CCI	F	30	1SGH	B	39
1MZW	B	31	6F4U	D	40
1OQE	K	31	2L9U	A	40
5KQ1	C	31	5OM2	B	40
5JPO	E	32	2XZE	R	40
2L34	A	33	4LZX	B	40
6B7G	B	33	5TUV	C	41
3MJH	B	34	2XA6	A	41
4NAW	D	34	2MOF	A	42
3DXC	B	35	2K9J	B	43
2XJY	B	35	2F9D	P	43
2BE6	D	37	4GDO	A	43
1IK9	C	37	6GNY	B	43
5XJL	M	37	6AU8	C	43
5FV8	E	38	2KS1	A	44
4UED	B	38	3HRO	A	44
5FV8	A	38	2L2T	A	44

**Table 2:** Amino acid properties.

Amino Acid	Hydrophobicity	Molecular Mass	Van der Waals Volume	Dipole Moment	Aromaticity	Acidity/Basicity
Glycine (G)	−0.4	57	48	0.000		
Alanine (A)	1.8	71	67	5.937		
Serine (S)	−0.8	87	73	9.836		
Proline (P)	−1.6	97	90	7.916		
Valine (V)	4.2	99	105	2.692		
Threonine (T)	−0.7	101	93	9.304		
Cysteine (C)	2.5	103	86	10.740		
Isoleucine (I)	4.5	113	124	3.371		
Leucine (L)	3.8	113	124	3.782		
Asparagine (N)	−3.5	114	96	18.890		
Aspartic acid (D)	−3.5	115	91	29.490		A
Glutamine (Q)	−3.5	128	114	39.890		
Lysine (K)	−3.9	128	135	50.020		B
Glutamic acid (E)	−3.5	129	109	42.520		A
Methionine (M)	1.9	131	124	8.589		
Histidine (H)	−3.2	137	118	20.440		B
Phenylalanine (F)	2.8	147	135	5.980	A	
Arginine (R)	−4.5	156	148	37.500		B
Tyrosine (Y)	−1.3	163	141	10.410	A	
Tryptophan (W)	−0.9	186	163	10.730	A	

**Table 3:** ProtBERT—categorical tests. The *p*-values correspond to the Mann–Whitney U test; *p*-values below 0.05 are considered significant and shown in boldface.

Method	Interactivity	Aromaticity	Acidity/Basicity	Interactivity	Aromaticity	Acidity/Basicity
	Embeddings—Target			Embeddings—Source		
Saliency	**6.09 × 10^−28^**	**1.87 × 10^−82^**	**8.98 × 10^−31^**	**1.88 × 10^−21^**	**1.21 × 10^−124^**	**7.03 × 10^−22^**
Deconvolution	5.05 × 10^−1^	2.83 × 10^−1^	2.79 × 10^−1^	**4.71 × 10^−2^**	**1.53 × 10^−4^**	2.55 × 10^−1^
Guided Backprop.	5.05 × 10^−1^	2.83 × 10^−1^	2.79 × 10^−1^	**4.71 × 10^−2^**	**1.53 × 10^−4^**	2.55 × 10^−1^
Input X Grad.	6.16 × 10^−2^	6.45 × 10^−1^	6.98 × 10^−1^	**2.41 × 10^−2^**	**1.86 × 10^−2^**	1.60 × 10^−1^
DeepLIFT	4.56 × 10^−1^	3.48 × 10^−1^	**1.47 × 10^−2^**	3.28 × 10^−1^	**1.62 × 10^−3^**	**2.15 × 10^−2^**
Integrated Grad.	5.09 × 10^−1^	9.09 × 10^−2^	**1.20 × 10^−8^**	**2.28 × 10^−19^**	**1.09 × 10^−5^**	**2.92 × 10^−19^**
LIME	3.82 × 10^−1^	5.47 × 10^−1^	3.93 × 10^−1^	6.28 × 10^−1^	3.55 × 10^−1^	2.24 × 10^−1^
KernelShap	**0.00 × 10^0^**	**3.74 × 10^−126^**	**2.41 × 10^−20^**	**3.95 × 10^−33^**	**7.09 × 10^−17^**	**1.01 × 10^−12^**
GradientShap	2.79 × 10^−1^	9.32 × 10^−1^	**8.37 × 10^−6^**	6.14 × 10^−1^	**2.35 × 10^−2^**	7.30 × 10^−1^
	Predictions—Target			Predictions—Source		
Saliency	**6.20 × 10^−52^**	**1.04 × 10^−170^**	**4.58 × 10^−38^**	7.43 × 10^−2^	**3.49 × 10^−219^**	**1.90 × 10^−32^**
Deconvolution	**1.65 × 10^−2^**	**1.29 × 10^−6^**	2.63 × 10^−1^	7.41 × 10^−1^	**6.66 × 10^−9^**	**8.29 × 10^−9^**
Guided Backprop.	**6.56 × 10^−3^**	9.32 × 10^−1^	4.12 × 10^−1^	2.53 × 10^−1^	**3.52 × 10^−3^**	3.53 × 10^−1^
Input X Grad.	9.01 × 10^−1^	8.19 × 10^−1^	7.53 × 10^−1^	2.88 × 10^−1^	**1.46 × 10^−2^**	4.90 × 10^−1^
DeepLIFT	**7.11 × 10^−13^**	4.81 × 10^−1^	3.41 × 10^−1^	**6.81 × 10^−6^**	**4.63 × 10^−4^**	7.06 × 10^−2^
Integrated Grad.	**6.86 × 10^−108^**	4.81 × 10^−1^	**4.23 × 10^−20^**	**1.51 × 10^−2^**	**9.82 × 10^−7^**	**2.76 × 10^−7^**
LIME	2.06 × 10^−1^	7.92 × 10^−1^	1.67 × 10^−1^	4.45 × 10^−1^	3.82 × 10^−1^	1.91 × 10^−1^
KernelShap	**3.96 × 10^−236^**	**4.68 × 10^−157^**	**1.12 × 10^−20^**	**2.49 × 10^−29^**	**2.97 × 10^−12^**	**1.34 × 10^−7^**
GradientShap	2.92 × 10^−1^	**2.87 × 10^−2^**	**4.41 × 10^−5^**	9.66 × 10^−1^	**2.82 × 10^−2^**	8.91 × 10^−1^

**Table 4:** ProtT5—categorical tests. The *p*-values correspond to the Mann–Whitney U test; *p*-values below 0.05 are considered significant and shown in boldface.

Method	Interactivity	Aromaticity	Acidity/Basicity	Interactivity	Aromaticity	Acidity/Basicity
	Embeddings—Target			Embeddings—Source		
Saliency	**2.58 × 10^−9^**	**5.67 × 10^−8^**	8.32 × 10^−2^	**2.49 × 10^−152^**	**1.19 × 10^−2^**	**8.39 × 10^−61^**
Deconvolution	**4.53 × 10^−15^**	7.06 × 10^−1^	**2.79 × 10^−2^**	**3.12 × 10^−2^**	**4.96 × 10^−45^**	**0.00 × 10^0^**
Guided Backprop.	**5.25 × 10^−6^**	9.70 × 10^−1^	**7.20 × 10^−60^**	**3.57 × 10^−14^**	**1.35 × 10^−35^**	**9.16 × 10^−139^**
Input X Grad.	6.85 × 10^−2^	7.12 × 10^−1^	4.75 × 10^−1^	3.25 × 10^−1^	9.18 × 10^−1^	3.60 × 10^−1^
DeepLIFT	5.95 × 10^−1^	3.73 × 10^−1^	5.07 × 10^−1^	**3.82 × 10^−22^**	**8.98 × 10^−30^**	**1.41 × 10^−21^**
Integrated Grad.	2.19 × 10^−1^	8.96 × 10^−2^	8.56 × 10^−1^	**9.56 × 10^−32^**	**4.23 × 10^−4^**	3.23 × 10^−1^
LIME	7.64 × 10^−1^	1.58 × 10^−1^	7.80 × 10^−1^	9.03 × 10^−1^	4.18 × 10^−1^	5.31 × 10^−1^
KernelShap	**1.17 × 10^−2^**	**3.79 × 10^−67^**	**5.89 × 10^−63^**	**2.84 × 10^−13^**	**3.61 × 10^−5^**	**6.75 × 10^−3^**
GradientShap	9.30 × 10^−2^	7.30 × 10^−2^	8.92 × 10^−1^	7.21 × 10^−2^	1.23 × 10^−1^	4.41 × 10^−1^
	Predictions—Target			Predictions—Source		
Saliency	5.61 × 10^−2^	**1.29 × 10^−33^**	2.52 × 10^−1^	**4.48 × 10^−132^**	6.03 × 10^−1^	**3.49 × 10^−127^**
Deconvolution	**8.16 × 10^−69^**	**5.95 × 10^−12^**	6.02 × 10^−1^	**1.71 × 10^−14^**	**9.20 × 10^−62^**	**0.00 × 10^0^**
Guided Backprop.	**5.26 × 10^−94^**	7.31 × 10^−2^	**8.84 × 10^−102^**	**9.26 × 10^−12^**	**1.17 × 10^−61^**	**9.02 × 10^−276^**
Input X Grad.	1.44 × 10^−1^	4.54 × 10^−1^	7.71 × 10^−1^	9.88 × 10^−1^	6.27 × 10^−1^	7.30 × 10^−1^
DeepLIFT	1.12 × 10^−1^	1.94 × 10^−1^	2.13 × 10^−1^	**2.27 × 10^−19^**	**1.67 × 10^−22^**	**2.37 × 10^−18^**
Integrated Grad.	**1.09 × 10^−2^**	8.95 × 10^−1^	9.01 × 10^−1^	**4.77 × 10^−28^**	8.94 × 10^−1^	9.82 × 10^−1^
LIME	8.42 × 10^−1^	1.42 × 10^−1^	7.55 × 10^−1^	6.80 × 10^−1^	6.38 × 10^−1^	7.21 × 10^−1^
KernelShap	**1.62 × 10^−93^**	**3.90 × 10^−7^**	**6.22 × 10^−21^**	**3.40 × 10^−6^**	**2.35 × 10^−3^**	**1.77 × 10^−3^**
GradientShap	5.14 × 10^−1^	7.91 × 10^−1^	2.08 × 10^−1^	7.09 × 10^−2^	3.88 × 10^−1^	9.76 × 10^−1^

**Table 5:** Ankh—categorical tests. The *p*-values correspond to the Mann–Whitney U test; *p*-values below 0.05 are considered significant and shown in boldface.

Method	Interactivity	Aromaticity	Acidity/Basicity	Interactivity	Aromaticity	Acidity/Basicity
	Embeddings—Target			Embeddings—Source		
Saliency	**5.59 × 10^−164^**	**2.64 × 10^−5^**	7.14 × 10^−2^	**9.23 × 10^−101^**	4.12 × 10^−1^	1.81 × 10^−1^
Deconvolution	**5.00 × 10^−3^**	8.79 × 10^−1^	**6.77 × 10^−16^**	**5.95 × 10^−22^**	7.93 × 10^−1^	**5.57 × 10^−7^**
Guided Backprop.	**5.00 × 10^−3^**	8.79 × 10^−1^	**6.77 × 10^−16^**	**5.95 × 10^−22^**	7.93 × 10^−1^	**5.57 × 10^−7^**
Input X Grad.	6.45 × 10^−1^	**3.69 × 10^−3^**	**6.14 × 10^−3^**	3.01 × 10^−1^	**5.36 × 10^−5^**	2.88 × 10^−1^
DeepLIFT	**5.75 × 10^−8^**	2.57 × 10^−1^	3.58 × 10^−1^	1.45 × 10^−1^	**2.65 × 10^−3^**	**2.80 × 10^−2^**
Integrated Grad.	2.50 × 10^−1^	**3.29 × 10^−2^**	**7.26 × 10^−8^**	**1.94 × 10^−27^**	**2.51 × 10^−87^**	**2.12 × 10^−288^**
LIME	2.18 × 10^−1^	2.40 × 10^−1^	3.04 × 10^−1^	**4.60 × 10^−2^**	9.19 × 10^−2^	6.05 × 10^−1^
KernelShap	**1.78 × 10^−21^**	**3.01 × 10^−3^**	**0.00 × 10^0^**	**1.61 × 10^−4^**	**1.54 × 10^−5^**	**1.04 × 10^−11^**
GradientShap	1.51 × 10^−1^	9.62 × 10^−1^	3.46 × 10^−1^	**5.43 × 10^−3^**	**5.03 × 10^−3^**	**2.45 × 10^−5^**
	Predictions—Target			Predictions—Source		
Saliency	**0.00 × 10^0^**	**1.70 × 10^−29^**	**8.82 × 10^−7^**	**2.22 × 10^−154^**	1.48 × 10^−1^	**7.43 × 10^−8^**
Deconvolution	**3.97 × 10^−14^**	**4.89 × 10^−5^**	**6.77 × 10^−8^**	**1.59 × 10^−15^**	**1.38 × 10^−12^**	5.22 × 10^−1^
Guided Backprop.	**5.68 × 10^−6^**	9.42 × 10^−2^	**1.99 × 10^−6^**	**1.38 × 10^−51^**	**4.74 × 10^−2^**	**2.40 × 10^−4^**
Input X Grad.	6.42 × 10^−1^	6.72 × 10^−2^	**1.75 × 10^−4^**	**4.05 × 10^−8^**	**1.51 × 10^−7^**	3.39 × 10^−1^
DeepLIFT	**1.39 × 10^−9^**	**1.93 × 10^−2^**	**8.31 × 10^−7^**	7.14 × 10^−2^	**8.33 × 10^−7^**	9.57 × 10^−1^
Integrated Grad.	1.19 × 10^−1^	9.03 × 10^−1^	**9.37 × 10^−3^**	**3.78 × 10^−15^**	**4.92 × 10^−62^**	1.30 × 10^−1^
LIME	2.02 × 10^−1^	2.04 × 10^−1^	1.02 × 10^−1^	7.91 × 10^−2^	9.86 × 10^−2^	5.28 × 10^−1^
KernelShap	**7.78 × 10^−11^**	7.89 × 10^−1^	**1.31 × 10^−213^**	**1.47 × 10^−2^**	**3.41 × 10^−3^**	**7.55 × 10^−9^**
GradientShap	1.17 × 10^−1^	8.57 × 10^−2^	5.02 × 10^−1^	**3.98 × 10^−4^**	**2.62 × 10^−9^**	**4.35 × 10^−2^**

**Table 6:** ProtBERT–numerical tests. Kendall correlations are presented as heat maps, with red and blue indicating positive and negative correlations, respectively; *p*-values are from the corresponding Kendall test for assessing whether the correlations are significantly different from zero; *p*-values below 0.05 are considered significant and shown in boldface.

Method	Hydrophobicity		Molecular Mass		Van Der Waals		Dipole Moment	
	Correlation	*p*-Value	Correlation	*p*-Value	Correlation	*p*-Value	Correlation	*p*-Value
	Embeddings—Target							
Saliency	0.300	0.068	−0.254	0.119	−0.021	0.896	−0.442	**0.006**
Deconvolution	−0.064	0.696	0.392	**0.016**	0.287	0.079	0.095	0.586
Guided Backprop.	−0.064	0.696	0.392	**0.016**	0.287	0.079	0.095	0.586
Input X Grad.	0.225	0.171	0.074	0.649	0.138	0.398	−0.147	0.386
DeepLIFT	0.182	0.268	−0.201	0.217	−0.170	0.298	−0.284	0.086
Integrated Grad.	−0.428	**0.009**	0.392	**0.016**	0.266	0.104	0.526	**0.001**
LIME	0.257	0.118	−0.180	0.269	−0.287	0.079	−0.200	0.233
KernelShap	−0.203	0.216	0.116	0.475	0.106	0.515	0.189	0.260
GradientShap	−0.171	0.297	0.243	0.135	0.192	0.242	0.221	0.186
	Embeddings—Source							
Saliency	0.278	0.090	−0.254	0.119	−0.043	0.795	−0.379	**0.020**
Deconvolution	0.118	0.473	−0.011	0.948	−0.106	0.515	0.137	0.422
Guided Backprop.	0.118	0.473	−0.011	0.948	−0.106	0.515	−0.137	0.422
Input X Grad.	0.000	1.000	−0.032	0.845	−0.106	0.515	−0.063	0.725
DeepLIFT	0.171	0.297	−0.201	0.217	−0.181	0.269	−0.179	0.288
Integrated Grad.	−0.182	0.268	−0.085	0.603	−0.266	0.104	0.232	0.165
LIME	−0.086	0.602	0.042	0.795	0.149	0.362	0.116	0.501
KernelShap	0.289	0.078	−0.169	0.299	0.064	0.696	−0.379	**0.020**
GradientShap	−0.011	0.948	−0.169	0.299	−0.106	0.515	0.126	0.461
	Predictions—Target							
Saliency	0.310	0.059	−0.212	0.194	0.000	1.000	−0.432	**0.007**
Deconvolution	−0.053	0.744	0.063	0.697	−0.149	0.362	0.147	0.386
Guided Backprop.	−0.300	0.068	0.042	0.795	−0.138	0.398	0.442	**0.006**
Input X Grad.	0.439	**0.008**	−0.085	0.603	−0.053	0.745	−0.432	**0.007**
DeepLIFT	0.203	0.216	−0.243	0.135	−0.160	0.329	−0.263	0.113
Integrated Grad.	−0.150	0.361	0.190	0.242	−0.043	0.795	0.337	**0.040**
LIME	0.503	**0.002**	−0.201	0.217	−0.170	0.298	−0.453	**0.005**
KernelShap	0.139	0.397	−0.085	0.603	0.000	1.000	−0.295	0.074
GradientShap	0.086	0.602	−0.243	0.135	−0.074	0.649	−0.084	0.631
	Predictions—Source							
Saliency	0.267	0.103	−0.265	0.104	−0.053	0.745	−0.389	**0.016**
Deconvolution	0.011	0.948	0.127	0.436	0.032	0.845	0.021	0.924
Guided Backprop.	−0.118	0.473	0.011	0.948	0.011	0.948	0.074	0.677
Input X Grad.	−0.096	0.557	−0.254	0.119	−0.383	**0.019**	−0.032	0.873
DeepLIFT	0.278	0.090	−0.254	0.119	−0.287	0.079	−0.284	0.086
Integrated Grad.	−0.524	**0.001**	0.360	**0.027**	0.213	0.193	0.495	**0.002**
LIME	−0.278	0.090	−0.127	0.436	−0.170	0.298	0.137	0.422
KernelShap	0.289	0.078	−0.180	0.269	0.053	0.745	−0.389	**0.016**
GradientShap	0.321	0.051	−0.190	0.242	0.011	0.948	−0.505	**0.001**

**Table 7:** ProtT5—numerical tests. Kendall correlations are presented as heat maps, with red and blue indicating positive and negative correlations, respectively; *p*-values are from the corresponding Kendall test for assessing whether the correlations are significantly different from zero; *p*-values below 0.05 are considered significant and shown in boldface.

Method	Hydrophobicity		Molecular Mass		Van Der Waals		Dipole Moment	
	Correlation	*p*-Value	Correlation	*p*-Value	Correlation	*p*-Value	Correlation	*p*-Value
	Embeddings—Target							
Saliency	0.246	0.134	−0.180	0.269	0.032	0.845	−0.368	**0.024**
Deconvolution	−0.278	0.090	0.169	0.299	−0.064	0.696	0.400	**0.014**
Guided Backprop.	0.257	0.118	−0.233	0.153	−0.032	0.845	−0.379	**0.020**
Input X Grad.	−0.011	0.948	−0.275	0.091	−0.383	**0.019**	−0.116	0.501
DeepLIFT	−0.214	0.192	0.201	0.217	0.053	0.745	0.274	0.098
Integrated Grad.	0.214	0.192	−0.021	0.897	−0.170	0.298	−0.211	0.209
LIME	−0.064	0.696	0.063	0.697	0.074	0.649	0.147	0.386
KernelShap	−0.492	**0.003**	0.370	**0.023**	0.223	0.172	0.695	**0.000**
GradientShap	−0.011	0.948	−0.106	0.516	−0.277	0.091	−0.053	0.773
	Embeddings—Source							
Saliency	0.364	**0.027**	−0.296	0.069	−0.064	0.696	−0.463	**0.004**
Deconvolution	−0.171	0.297	0.339	**0.038**	0.106	0.515	0.295	0.074
Guided Backprop.	0.342	**0.037**	−0.106	0.516	0.021	0.896	−0.389	**0.016**
Input X Grad.	−0.107	0.514	−0.063	0.697	−0.106	0.515	0.084	0.631
DeepLIFT	0.000	1.000	0.381	**0.019**	0.277	0.091	0.074	0.677
Integrated Grad.	0.182	0.268	0.021	0.897	0.064	0.696	−0.063	0.725
LIME	0.193	0.241	0.063	0.697	0.074	0.649	−0.095	0.586
KernelShap	−0.267	0.103	0.180	0.269	−0.053	0.745	0.389	**0.016**
GradientShap	−0.171	0.297	0.180	0.269	0.032	0.845	0.326	**0.047**
	Predictions—Target							
Saliency	0.214	0.192	−0.222	0.173	−0.011	0.948	−0.368	**0.024**
Deconvolution	−0.300	0.068	0.212	0.194	0.011	0.948	0.442	**0.006**
Guided Backprop.	0.396	**0.016**	−0.159	0.330	0.011	0.948	−0.463	**0.004**
Input X Grad.	0.214	0.192	−0.169	0.299	−0.106	0.515	−0.326	**0.047**
DeepLIFT	0.342	**0.037**	−0.095	0.559	0.053	0.745	−0.189	0.260
Integrated Grad.	−0.182	0.268	0.349	**0.032**	0.160	0.329	0.263	0.113
LIME	−0.385	**0.019**	0.254	0.119	0.149	0.362	0.337	**0.040**
KernelShap	0.257	0.118	−0.392	**0.016**	−0.287	0.079	−0.421	**0.009**
GradientShap	−0.064	0.696	0.254	0.119	0.032	0.845	0.095	0.586
	Predictions—Source							
Saliency	0.364	**0.027**	−0.296	0.069	−0.064	0.696	−0.463	**0.004**
Deconvolution	−0.171	0.297	0.339	**0.038**	0.106	0.515	0.295	0.074
Guided Backprop.	0.342	**0.037**	−0.106	0.516	0.021	0.896	−0.389	**0.016**
Input X Grad.	0.449	**0.006**	−0.265	0.104	−0.170	0.298	−0.579	**0.000**
DeepLIFT	0.021	0.896	−0.021	0.897	0.053	0.745	0.000	1.000
Integrated Grad.	−0.118	0.473	−0.127	0.436	−0.255	0.118	0.084	0.631
LIME	0.000	1.000	0.042	0.795	0.021	0.896	−0.179	0.288
KernelShap	0.278	0.090	−0.222	0.173	0.032	0.845	−0.368	**0.024**
GradientShap	−0.043	0.794	0.063	0.697	0.021	0.896	0.116	0.501

**Table 8:** Ankh—numerical tests. Kendall correlations are presented as heat maps, with red and blue indicating positive and negative correlations, respectively; *p*-values are from the corresponding Kendall test for assessing whether the correlations are significantly different from zero; *p*-values below 0.05 are considered significant and shown in boldface.

Method	Hydrophobicity		Molecular Mass		Van Der Waals		Dipole Moment	
	Correlation	*p*-Value	Correlation	*p*-Value	Correlation	*p*-Value	Correlation	*p*-Value
	Embeddings—Target							
Saliency	0.278	0.090	−0.212	0.194	0.000	1.000	−0.358	**0.028**
Deconvolution	−0.353	**0.031**	0.042	0.795	−0.085	0.603	0.347	**0.034**
Guided Backprop.	−0.353	**0.031**	0.042	0.795	−0.085	0.603	0.347	**0.034**
Input X Grad.	0.160	0.328	−0.116	0.475	−0.011	0.948	−0.095	0.586
DeepLIFT	0.300	0.068	−0.254	0.119	−0.032	0.845	−0.295	0.074
Integrated Grad.	−0.289	0.078	0.021	0.897	−0.149	0.362	0.326	**0.047**
LIME	−0.214	0.192	0.127	0.436	0.043	0.795	0.063	0.725
KernelShap	0.011	0.948	−0.127	0.436	−0.021	0.896	−0.168	0.319
GradientShap	−0.203	0.216	0.063	0.697	−0.021	0.896	0.137	0.422
	Embeddings—Source							
Saliency	0.225	0.171	−0.307	0.060	−0.096	0.558	−0.326	**0.047**
Deconvolution	−0.182	0.268	0.074	0.649	0.011	0.948	0.295	0.074
Guided Backprop.	−0.182	0.268	0.074	0.649	0.011	0.948	0.295	0.074
Input X Grad.	0.021	0.896	0.233	0.153	0.362	**0.027**	0.000	1.000
DeepLIFT	0.075	0.648	0.021	0.897	0.181	0.269	−0.179	0.288
Integrated Grad.	−0.246	0.134	−0.159	0.330	−0.330	**0.044**	0.179	0.288
LIME	0.128	0.434	−0.169	0.299	−0.170	0.298	−0.116	0.501
KernelShap	0.246	0.134	−0.212	0.194	0.043	0.795	−0.358	**0.028**
GradientShap	−0.235	0.152	−0.180	0.269	−0.298	0.069	0.200	0.233
	Predictions—Target							
Saliency	0.257	0.118	−0.212	0.194	−0.011	0.948	−0.400	**0.014**
Deconvolution	−0.257	0.118	0.201	0.217	0.053	0.745	0.358	**0.028**
Guided Backprop.	−0.246	0.134	0.159	0.330	−0.085	0.603	0.389	**0.016**
Input X Grad.	0.203	0.216	0.201	0.217	0.192	0.242	−0.053	0.773
DeepLIFT	0.075	0.648	−0.074	0.649	−0.149	0.362	0.074	0.677
Integrated Grad.	0.289	0.078	−0.063	0.697	−0.064	0.696	−0.253	0.128
LIME	0.385	**0.019**	−0.455	**0.005**	−0.383	**0.019**	−0.442	**0.006**
KernelShap	0.257	0.118	−0.085	0.603	−0.181	0.269	−0.253	0.128
GradientShap	0.118	0.473	0.169	0.299	0.149	0.362	0.042	0.823
	Predictions—Source							
Saliency	0.257	0.118	−0.317	0.051	−0.106	0.515	−0.358	**0.028**
Deconvolution	−0.193	0.241	0.053	0.745	−0.011	0.948	0.274	0.098
Guided Backprop.	−0.439	**0.008**	0.159	0.330	0.032	0.845	0.505	**0.001**
Input X Grad.	−0.160	0.328	−0.063	0.697	−0.213	0.193	0.242	0.146
DeepLIFT	−0.075	0.648	−0.053	0.745	−0.223	0.172	0.116	0.501
Integrated Grad.	0.021	0.896	0.085	0.603	0.234	0.152	0.000	1.000
LIME	0.043	0.794	−0.127	0.436	−0.043	0.795	0.032	0.873
KernelShap	−0.075	0.648	0.085	0.603	−0.128	0.435	0.137	0.422
GradientShap	0.417	**0.011**	0.042	0.795	0.074	0.649	−0.189	0.260

**Table 9:** Overall number of tests passed. Colour map added for readability; darker colours correspond to higher numbers.

Embedding	ProtBERT			ProtT5			Ankh			Total by XAI		
XAI Method	Cat.	Num.	Tot.	Cat.	Num.	Tot.	Cat.	Num.	Tot.	Cat.	Num.	Tot.
ine Saliency	11	4	15	8	6	14	8	4	12	27	14	41
Deconvolution	6	1	7	10	4	14	9	3	12	25	8	33
Guided Backprop.	4	2	6	10	7	17	9	5	14	23	14	37
Input X Grad.	3	3	6	0	4	4	6	1	7	9	8	17
DeepLIFT	6	0	6	6	2	8	7	0	7	19	2	21
Integrated Grad.	9	7	16	4	1	5	8	2	10	21	10	31
LIME	0	2	2	0	2	2	1	4	5	1	8	9
KernelShap	12	2	14	12	7	19	11	1	12	35	10	45
GradientShap	5	1	6	0	1	1	6	1	7	11	3	14
ine Total by Embed.	56	22	78	50	34	84	65	21	86	171	77	248

**Table 10:** Tests passed by XAI methods and test type.

XAI Method	Target	Source	Embedding	Prediction
Saliency	20	21	21	20
Deconvolution	17	16	16	17
Guided Backpropagation	17	20	17	20
Input X Gradient	7	10	7	10
DeepLIFT	7	14	10	11
Integrated Gradient	13	18	16	15
LIME	8	1	1	8
KernelShap	22	23	24	21
GradientShap	3	11	6	8

**Table 11:** Tests passed by embedding and test type.

Embedding	ProtBERT			ProtT5			Ankh			Total by Test Type		
Test Type	Cat.	Num.	Total	Cat.	Num.	Total	Cat.	Num.	Total	Cat.	Num.	Total
Target	23	13	36	18	18	36	29	13	42	70	44	114
Source	33	9	42	32	16	48	36	8	44	101	33	134
Embedding	27	8	35	26	15	41	32	10	42	85	33	118
Prediction	29	14	43	24	19	43	33	11	44	86	44	130

**Table 12:** Comparison of embedding vs. prediction in terms of test passing.

Embedding	ProtBERT			ProtT5			Ankh			All		
Embed.|Predict.	Cat.	Num.	Total	Cat.	Num.	Total	Cat.	Num.	Total	Cat.	Num.	Total
pass|pass	21	4	25	22	11	33	25	4	29	68	19	87
pass|fail	6	4	10	4	4	8	7	6	13	17	14	31
fail|pass	8	10	18	2	8	10	8	7	15	18	25	43
fail|fail	19	54	73	26	49	75	14	55	69	59	158	217
Total	54	72	126	54	72	126	54	72	126	162	216	378

**Table 13:** Mean infidelity results with a separate heat map for each column; darker is better (lower infidelity); colors in different columns are not comparable.

Mean Infidelity	ProtBERT		ProtT5		Ankh	
XAI Method	Embed.	Predict.	Embed.	Predict.	Embed.	Predict.
ine Saliency	6.98 × 10^−8^	5.03 × 10^−5^	5.76 × 10^−9^	5.50 × 10^−6^	2.05 × 10^−11^	3.58 × 10^−6^
Deconvolution	7.03 × 10^−8^	5.31 × 10^−5^	7.64 × 10^−9^	6.98 × 10^−6^	2.03 × 10^−11^	6.17 × 10^−5^
Guided Backprop.	6.95 × 10^−8^	4.93 × 10^−5^	1.14 × 10^−1^	1.10 × 10^−4^	2.02 × 10^−11^	1.63 × 10^−6^
Input X Gradient	5.15 × 10^−8^	3.73 × 10^−5^	6.49 × 10^−9^	4.75 × 10^−6^	4.81 × 10^−10^	2.09 × 10^−6^
DeepLIFT	4.61 × 10^−8^	3.32 × 10^−5^	8.42 × 10^−8^	7.90 × 10^−5^	5.80 × 10^−10^	2.08 × 10^−6^
Integrated Gradient	4.27 × 10^−8^	3.22 × 10^−5^	4.10 × 10^−9^	3.50 × 10^−6^	1.36 × 10^−11^	1.69 × 10^−6^
LIME	4.40 × 10^−8^	3.22 × 10^−5^	2.39 × 10^−7^	3.51 × 10^−6^	4.34 × 10^−11^	1.77 × 10^−6^
KernelShap	4.47 × 10^−8^	3.25 × 10^−5^	1.00 × 10^−6^	1.00 × 10^−6^	1.61 × 10^−11^	1.74 × 10^−6^
GradientShap	4.56 × 10^−8^	3.03 × 10^−5^	4.52 × 10^−8^	4.21 × 10^−5^	3.10 × 10^−10^	2.03 × 10^−6^

**Table 14:** Tests passed by XAI methods and embeddings, with the same organization as in Table 13 for the mean infidelity results; darker is better.

Embedding	ProtBERT		ProtT5		Ankh	
XAI Method	Embed.	Predict.	Embed.	Predict.	Embed.	Predict.
ine Saliency	8	7	8	6	5	7
Deconvolution	3	4	7	7	6	6
Guided Backprop.	3	3	8	9	6	8
Input X Grad.	2	4	1	3	4	3
DeepLIFT	3	3	4	4	3	4
Integrated Grad.	7	9	2	3	7	3
LIME	0	2	0	2	1	4
KernelShap	7	7	10	9	7	5
GradientShap	2	4	1	0	3	4

## Data Availability

The software used for this project is available at github.com/lucian-ilie/Protein-XAI (accessed on 25 May 2025).
